# Exploring the interactive effect of dysfunctional sleep beliefs and mental health on sleep in university students

**DOI:** 10.3389/frsle.2024.1340729

**Published:** 2024-03-28

**Authors:** Sameena Karsan, Tara Kuhn, Michelle Ogrodnik, Laura E. Middleton, Jennifer J. Heisz

**Affiliations:** ^1^Neurofit Lab, Department of Kinesiology, McMaster University, Hamilton, ON, Canada; ^2^Department of Kinesiology and Health Sciences, University of Waterloo, Waterloo, ON, Canada

**Keywords:** sleep beliefs, insomnia, university, stress, mental health, people of color

## Abstract

**Introduction:**

Poor mental health is a known risk factor for poor sleep among university students; however, less is known about the role of dysfunctional sleep beliefs and its relation to mental health and sleep. Additionally, students who identify as people of color (POC) may experience unique stressors related to discrimination and inequalities which can contribute to mental health issues and in turn, influence their sleep. The present study evaluated the impact of dysfunctional sleep beliefs and poor mental health on a student's susceptibility to worse sleep and examined differences among POC.

**Methods:**

Post-secondary students completed a survey including the Insomnia Severity Index and the Dysfunctional Beliefs and Attitudes about Sleep Scale. Participants also completed questionnaires measuring symptoms of depression, anxiety, and perceived stress.

**Results:**

One thousand five hundred and sixty-two students were included in the analyses, 58% of which were POC. POC students had more dysfunctional sleep beliefs (*p* < 0.01) and worse insomnia severity (*p* < 0.01) compared to white students. Overall, greater dysfunctional sleep beliefs were significantly associated with worse symptoms of depression (*b* = 1.521), anxiety (*b* = 1.170), stress (*b* = 1.370), and poor sleep (*b* =1.963; *ps* < 0.001). Dysfunctional sleep beliefs also moderated the relation between poor mental health and sleep, specifically depression (*p* = 0.035) and anxiety (*p* = 0.007), by exacerbating sleep outcomes.

**Discussion:**

The results suggest that dysfunctional sleep beliefs may play a role in perpetuating poor mental health and sleep. Interventions to improve students' sleep and wellbeing focused on reframing dysfunctional sleep beliefs should be examined.

## 1 Introduction

Sleep is vital for mental health (Fernandez-Mendoza and Vgontzas, 2013). Yet ~20% of university students experience symptoms of insomnia and therefore, may not be getting adequate sleep for good health (Jiang et al., 2015). This is harmful as a single night of insufficient sleep can cause daytime sleepiness, mood disruptions, and cognitive impairments (Aguirre, 2016). Although some students are more impacted than others, the factors elevating their risk of poor sleep and poor mental health remain unclear (Sheldon et al., 2021). The present study sought to identify psychosocial factors influencing students' susceptibility to poor sleep and mental health.

A person's undergraduate degree is a pivotal point in their lives, and many are tasked with academic and extracurricular workloads that place exceeding demands on their mental and physical wellbeing (Laidlaw et al., 2016). It is estimated that 60% of students suffer from one or more mental health problems (Lipson et al., 2022). Chronically high levels of psychological stress increase the risk of poor mental health, and can directly impact sleep, which can lead to symptoms of insomnia (Yang et al., 2014; Pascoe et al., 2020). Everyday stressors such as academic and social pressures make it more difficult for students to maintain good sleep habits (Shaw et al., 2017; Reddy et al., 2018) and can elevate stress hormones like cortisol that decrease sleep quality and quantity (Porkka-Heiskanen et al., 2013).

Most research relating stress with sleep in students has either included white students only or has failed to report statistics for people of color (POC; Charles et al., 2011; Eskildsen et al., 2017). The few studies that report on POC suggest that POC have greater mental distress but lower rates of mental health service utilization than their peers (Lipson et al., 2022). Although the reasons are multifaceted, one explanation may be that racialized people experience a weakened sense of belonging to local communities (Chiu et al., 2018), and the discrimination and other unique stressors (e.g., cultural mindsets) they face have the potential to negatively impact their sleep by shortening durations and reducing sleep quality (Yip, 2015; Eliasson et al., 2017). Consequently, race and ethnicity are important variables to consider when evaluating the relationship between mental health and sleep in students.

Counterproductive or distorted thoughts about sleep may be another risk factor for mental illness among students, especially POC, but the impact of sleep beliefs on mental health has not yet been fully investigated. One way that dysfunctional sleep beliefs could worsen mental health and subsequently sleep is by potentiating perceived stress. For example, dysfunctional sleep beliefs may manifest as unrealistic expectations about sleep (e.g., “Without adequate sleep I will not be able to function well the next day” or “I must catch up on sleep loss”) and could give rise to stress-provoking feelings of hopelessness or helplessness, which are commonly associated with depression (Morin et al., 2007; Humphries et al., 2022). Dysfunctional sleep beliefs may also cause fearful anticipation and avoidance behaviors, which are known to augment perceived stress and elevate anxiety (Grupe and Nitschke, 2013). Although prior research demonstrates an association between dysfunctional sleep beliefs and insomnia severity (Jansson-Fröjmark and Linton, 2008; Eidelman et al., 2016; Chang et al., 2020), these studies primarily focus on clinical populations (e.g., psychiatric outpatients) or older adults. The studies that include young adults only report on sleep quality rather than its relation to mental health (Yang et al., 2011; Humphries et al., 2022). Therefore, it remains unclear whether a student's dysfunctional sleep beliefs impact their sleep and poor mental health simultaneously.

The present study examined the impact of dysfunctional sleep beliefs on mental health and sleep in a diverse sample of students. We first examined whether sleep and mental health outcomes differed between POC and white students. We then assessed the association between dysfunctional sleep beliefs, mental health, and sleep outcomes and predicted that all variables would be related to one another. We also tested whether dysfunctional sleep beliefs negatively impact mental health and whether dysfunctional sleep beliefs worsen health outcomes by exacerbating the negative impact of poor mental health on sleep. We hypothesize that the relationship between dysfunctional sleep beliefs and mental health is interdependent, and dysfunctional sleep beliefs may be associated with worse mental health issues and more disturbed sleep. The current research is increasingly important as there is a growing mental health crisis among students (Lipson et al., 2022) and the complex interplay between contributing factors needs to be examined so that more inclusive and accessible supports can be created to prevent maladaptive outcomes.

## 2 Methods

### 2.1 Participants

This cross-sectional study was part of a larger survey examining the relationship between sleep attitudes and beliefs in university students. To maximize the sample size, the survey was distributed in March 2022 at two Ontario universities. Participants were deemed eligible if they were currently enrolled as undergraduate or graduate students at McMaster University or the University of Waterloo and, after providing consent, completed the online survey. The study was approved by the ethics board of McMaster University (MREB #5834) and the University of Waterloo (ORE #43903).

McMaster University had approximately 37,000 eligible students, and the University of Waterloo had about 42,500 (University of Waterloo, 2018; McMaster University, 2022a). At McMaster, about 21% of students are international, and roughly 19% identify as racialized individuals (McMaster University, 2022b). At the University of Waterloo, 68% identify as visible minorities (University of Waterloo, 2018).

### 2.2 Measures

Participants reported demographics, including using an open-ended question to self-describe their ethnicity and whether they had ever been diagnosed with a psychiatric disorder. Since participants gave a range of answers for their ethnicity, responses were categorized using the racial and ethnic categories as described by Public Health Ontario (Black, East Asian, Latino, Middle Eastern, South Asian, Southeast Asian, Indigenous, White, or other; Public Health Ontario, 2021). The variable was labeled as “race/ethnicity” for all analyses. Participants were then split into one of two groups: white and POC.

Sleep beliefs were assessed using the Dysfunctional Beliefs and Attitudes about Sleep scale (DBAS-16) which is a validated tool used frequently to assess both clinical and non-clinical populations (Morin et al., 2007). The DBAS is a 16-item self-report measure that evaluates maladaptive cognitions about sleep. This measure assesses five domains including expectations of sleep requirements, effects of insomnia, sleep medication, worry and helplessness about insomnia, and global dysfunctional beliefs (Morin et al., 2007). Each statement is rated using a scale of 0 to 10 where 0 = “Strongly Disagree” and 10 = “Strongly Agree.” The total score is then calculated by averaging all answers, with higher averages indicating more severe dysfunctional beliefs and attitudes about sleep. The DBAS has demonstrated good internal consistency (Cronbach's alpha = 0.80; Morin et al., 2007) and has been tested in university student samples like the present study (Castillo et al., 2023).

The Insomnia Severity Index (ISI) was used to screen for poor sleep and measure symptoms of insomnia. The ISI is a valid measure that demonstrates good internal consistency (Cronbach's alpha = 0.83; Cerri et al., 2023) and has been used as both a screening and outcome measurement tool in clinical and non-clinical samples, including post-secondary students (Morin, 1993; Lukowski and Tsukerman, 2021). The ISI consists of seven items assessing the severity of insomnia problems that are graded on a scale from 0 to 4 (none = 0, mild = 1, moderate = 2, severe = 3, very = 4). The total score is then calculated through the sum of answers given, with a higher score indicating greater insomnia severity and poorer sleep outcomes. Given the total score, respondents are placed in one of four categories from “No clinically significant insomnia” to “Severe clinical insomnia.”

Depressive symptoms were assessed using the Patient Health Questionnaire-9 (PHQ). The 9-item questionnaire has been used to determine depressive symptoms in primary care and research settings (Spitzer et al., 1999). Each question is answered using a graded scale from 0 to 3 where a higher number indicates increased depression severity (not at all = 0, several days = 1, more than half the days = 2, nearly every day = 3). All answers are summed for a total score, and a higher total indicates greater severity of depressive symptoms. The PHQ-9 has been widely used to screen for depression in students and is recommended when assessing university students (Zhang, 2020). This tool has excellent internal consistency with studies reporting Cronbach's alpha close to 0.90 (Sun et al., 2020).

The Generalized Anxiety Disorder-7 (GAD-7) was used to screen participants for generalized anxiety disorder severity (Spitzer et al., 2006). This measure has been deemed reliable and valid, with good internal consistency as demonstrated by Cronbach's alpha ranging from 0.88 to 0.94 (Alghadir et al., 2020). This tool is often used in primary care and research settings involving students (Löwe et al., 2017; Dhira et al., 2021). The seven items of the questionnaire are rated on a scale of 0 to 3 with increasing severity (not at all = 0, several days = 1, more than half the days = 2, nearly every day = 3). All answers are summed to provide a total score, and increased severity is indicated by a higher score.

The Perceived Stress Scale (PSS) was used to measure an individual's level of perceived stress (Cohen et al., 1994). The 10-item questionnaire was graded on a scale from 0 to 4 with increasing severity of symptoms (never = 0, almost never = 1, sometimes = 2, fairly often = 3, very often = 4). The total score was calculated through the sum of answers with a higher score indicating increased perceived stress severity. The PSS is a validated questionnaire with a Cronbach's alpha between 0.60 to 0.82 (Anwer et al., 2020), and has been evaluated to have sufficient internal consistency and reliability among university students (Roberti et al., 2006).

### 2.3 Statistical analysis

Analyses were conducted in IBM SPSS Statistics Version 28. Normality and homoscedasticity were assessed through visual inspection and the Kolmogorov-Smirnov test. For all analyses, an alpha level of 0.05 was used to determine statistical significance. Independent samples *t*-test were used to explore differences in dysfunctional sleep beliefs, insomnia severity, perceived stress, and mental health between white and POC respondents. The association between dysfunctional sleep beliefs, perceived stress, insomnia severity, and mental health outcomes were assessed using two-tailed Pearson correlations.

Linear regression analyses were then used to test the independent contribution of dysfunctional sleep beliefs on mental health outcomes. The baseline model included age, biological sex, income, diagnosed psychological disorders, and race/ethnicity (white vs. POC) as these variables relate to mental health (Orgeta, 2009; Sareen et al., 2011; Altemus et al., 2014) and sleep beliefs (Ruggiero et al., 2019; Chang et al., 2020). Hayes PROCESS Macro, Version 4.1 was used for moderation analyses (Model 1) to test the effect of dysfunctional sleep beliefs on sleep and whether dysfunctional sleep beliefs moderate the relationship between mental health outcomes and sleep (Hayes, 2022). Due to the highly prevalent co-occurrence of depression, anxiety, and perceived stress (Lallukka et al., 2019), all variables were controlled for in each model. As an exploratory step, we conducted moderation analyses with race/ethnicity as a grouping variable to see if results differed for students who identified as white vs. POC.

Data were initially screened for extreme and missing values. Participants with less than half of the survey filled out or those who had extreme values for self-reported questions (i.e., inserted random values outside of accepted values) were removed (0.1%). Mental health and sleep outcomes were screened for missingness. Participants with two or more missing values for the mental health and sleep questionnaires were removed (0.4%); the remaining missing values were imputed (0.5%) using mean imputation based on the respondent's provided answers (Baraldi and Enders, 2010).

## 3 Results

### 3.1 Demographics

A total of 1,570 participants completed the survey. After assessing extreme and missing data, 1,568 were eligible respondents. Additionally, six participants were excluded due to missing mental health data, for a final sample size of 1,562. The average age of participants was 21.8 years. Over half of the participants were from McMaster University and 72% were female. Forty-two percent of respondents identified as white and 58% identified as POC or other. Of the POC, 36% identified as South Asian, 32% as East Asian, 6% as Black, 6% as Middle Eastern, 2% were Latino, 2% Southeast Asian, 0.4% were Indigenous, and 16% identified as multiracial or other. The descriptive characteristics of participants are displayed in Table 1.

**Table 1:** Demographic characteristics.

**Demographic characteristics**	**Variable**	**White students**	**POC students**
		***N*** **(%)**	***N*** **(%)**
School	McMaster University	351 (54)	482 (53)
	University of Waterloo	303 (46)	426 (47)
Sex	Female	496 (76)	626 (69)
	Male	153 (23)	277 (30)
	NA	5 (1)	5 (1)
Income	< $20,000	121 (19)	146 (16)
	$20,000–$49,999	100 (15)	207 (23)
	$50,000–$99,999	161 (24)	237 (26)
	>$100,000	254 (39)	293 (32)
	NA	18 (3)	25 (3)
PHQ-9 severity	None/minimal (1–4)	164 (25)	230 (25)
	Mild (5–9)	214 (33)	277 (30)
	Moderate (10–19)	145 (22)	178 (20)
	Severe (20–27)	131 (20)	223 (25)
	*Average (± SD)*	*9.28 (± 6.05)*	*9.70 (± 6.56)*
GAD-7 severity	None/minimal (0–4)	159 (24)	224 (25)
	Mild (5–9)	205 (31)	263 (29)
	Moderate (10–14)	165 (25)	247 (27)
	Severe (≥15)	125 (20)	174 (19)
	*Average (± SD)*	*9.16 (± 5.56)*	*9.13 (± 5.77)*
PSS severity	None/minimal (0–13)	96 (14)	122 (13)
	Mild	-	-
	Moderate (14–26)	430 (66)	596 (66)
	Severe (27–40)	128 (20)	190 (21)
	*Average (± SD)*	*20.62 (± 6.71)*	*21.04 (± 6.70)*
ISI severity	Not clinically significant (0–7)	272 (42)	339 (37)
	Subthreshold insomnia (8–14)	265 (40)	358 (40)
	Clinical insomnia (15–21)	109 (17)	181 (20)
	Severe clinical insomnia (22–28)	8 (1)	30 (3)
	*Average (± SD)*	*9.20 (± 5.55)*	10.05 (± 5.86)^**^
DBAS-16	*Average Score (± SD)*	*4.57 (± 1.44)*	4.79 (± 1.57)^**^

POC, Persons of color; PHQ-9, patient health questionnaire (depression); GAD-7, generalized anxiety disorder 7-item scale (anxiety); PSS, perceived stress scale (stress); ISI, insomnia severity index; DBAS, dysfunctional beliefs and attitudes about sleep scale.

^**^p < 0.01 for t-tests between groups.

Twenty-one percent of participants reported insomnia symptoms that met or exceeded clinical criteria for the disorder, indicating overall poor sleep and 86% of all students reported moderate to severe perceived stress levels. Average depressive (9.53 ± 6.35) and anxiety (9.14 ± 5.68) symptoms were within the mild-to-moderate range of symptom severity. However, 23% of students met the cut-off for severe depressive symptoms and 19% for severe symptoms of anxiety. Group differences revealed that POC students reported having significantly more dysfunctional sleep beliefs [*t*_(1,560)_ = −2.80, *p* < 0.01] and insomnia severity [*t*_(1,560)_ = −2.73, *p* < 0.01] compared to white students (Table 1).

Correlational analyses revealed that all variables were significantly related (Table 2). More severe dysfunctional sleep beliefs, perceived stress, depression, and anxiety symptoms were all associated with one another and to insomnia symptom severity (all *p*s < 0.001).

**Table 2:** Pearson's correlations between mental health, insomnia, and sleep beliefs.

	**ISI**	**DBAS-16**	**PSS**	**GAD-7**	**PHQ-9**
ISI	-	-	-	-	-
DBAS-16	0.54^***^	-	-	-	-
PSS	0.55^***^	0.35^***^	-	-	-
GAD-7	0.56^***^	0.35^***^	0.76^***^	-	-
PHQ-9	0.69^***^	0.41^***^	0.72^***^	0.73^***^	-

ISI, insomnia severity index; DBAS, dysfunctional beliefs and attitudes about sleep; PSS, perceived stress scale; GAD, generalized anxiety disorder; PHQ, patient health questionnaire.

^***^p < 0.001.

### 3.2 Impact of dysfunctional sleep beliefs on mental health and sleep

For depressive symptoms, the baseline model (age, biological sex, income, previous psychiatric diagnosis, and race/ethnicity) explained 11% of the variance [*R*^2^ = 0.115, *F*_(5,1,508)_ = 39.205, *p* < 0.001]. Adding dysfunctional sleep beliefs significantly improved the model and explained an additional 13% of the variance [*R*^2^ = 0.242, *F*_(6,1,507)_ = 80.274, *p* < 0.001]. When examining coefficients independently, higher dysfunctional sleep beliefs predicted worse depressive symptoms (*b* = 1.521; *p* < 0.001). A similar pattern was observed for anxiety, and perceived stress. The baseline models explained 10% variance for anxiety [*R*^2^ = 0.099, *F*_(5,1,508)_ = 32.989, *p* < 0.001] and perceived stress [*R*^2^ = 0.101, *F*_(5,1,508)_ = 34.021, *p* < 0.001]. The models were significantly improved by dysfunctional sleep beliefs. Similar to depressive symptoms, higher dysfunctional sleep beliefs predicted greater anxiety (*b* = 1.170, *p* < 0.001) and perceived stress (*b* = 1.370; *p* < 0.001). The covariates of age, sex, and previous psychological diagnosis had a significant effect on all models (*p*s < 0.001; Table 3).

**Table 3:** Linear regression values for dysfunctional sleep beliefs (DBAS) predicting mental health outcomes.

**Outcomes**	***R^2^* ^††^**	***ΔR^2^***	***b* ^††^**	***SE b* ^††^**	**β^††^**	***p* ^††^**
**Depression**	0.242					
Age		0.115^†^	−0.172	0.030	−0.132	< 0.001
Sex			−0.983	0.313	−0.071	0.002
Income			−0.199	0.130	−0.035	0.127
Psychiatric diagnosis			−2.587	0.255	−0.232	< 0.001
Race/ethnicity			−0.094	0.057	−0.037	0.097
DBAS		0.127^††^	1.521	0.096	0.363	< 0.001
**Anxiety**	0.192					
Age		0.099^†^	−0.154	0.027	−0.132	< 0.001
Sex			−1.777	0.289	−0.143	< 0.001
Income			−0.024	0.120	−0.005	0.842
Psychiatric diagnosis			−1.786	0.236	−0.179	< 0.001
Race/ethnicity			−0.062	0.052	−0.027	0.237
DBAS		0.094^††^	1.170	0.088	0.312	< 0.001
**Perceived stress**	0.194					
Age		0.101^†^	−0.183	0.032	−0.133	< 0.001
Sex			−2.033	0.341	−0.139	< 0.001
Income			−0.049	0.142	−0.008	0.728
Psychiatric diagnosis			−2.201	0.278	−0.187	< 0.001
Race/ethnicity			−0.094	0.062	−0.035	0.129
DBAS		0.092^††^	1.370	0.104	0.309	< 0.001
**Sleep**	0.315					
Age		0.056^†^	−0.070	0.049	−1.435	0.151
Sex			−0.102	0.112	−0.913	0.362
Income			−0.482	0.269	−1.791	0.073
Psychiatric diagnosis			−1.327	0.220	−6.042	< 0.001
Race/ethnicity			−0.040	0.026	−1.565	0.118
DBAS		0.259^††^	1.963	0.082	23.848	< 0.001

Sex, income, psychiatric diagnosis, and race/ethnicity were used as covariates.

DBAS, dysfunctional beliefs and attitudes about sleep.

^†^Baseline model (age, biological sex, income, diagnosed psychological disorders, and race/ethnicity).

^††^Full model containing DBAS scores in addition to baseline predictors.

**Figure 1:**
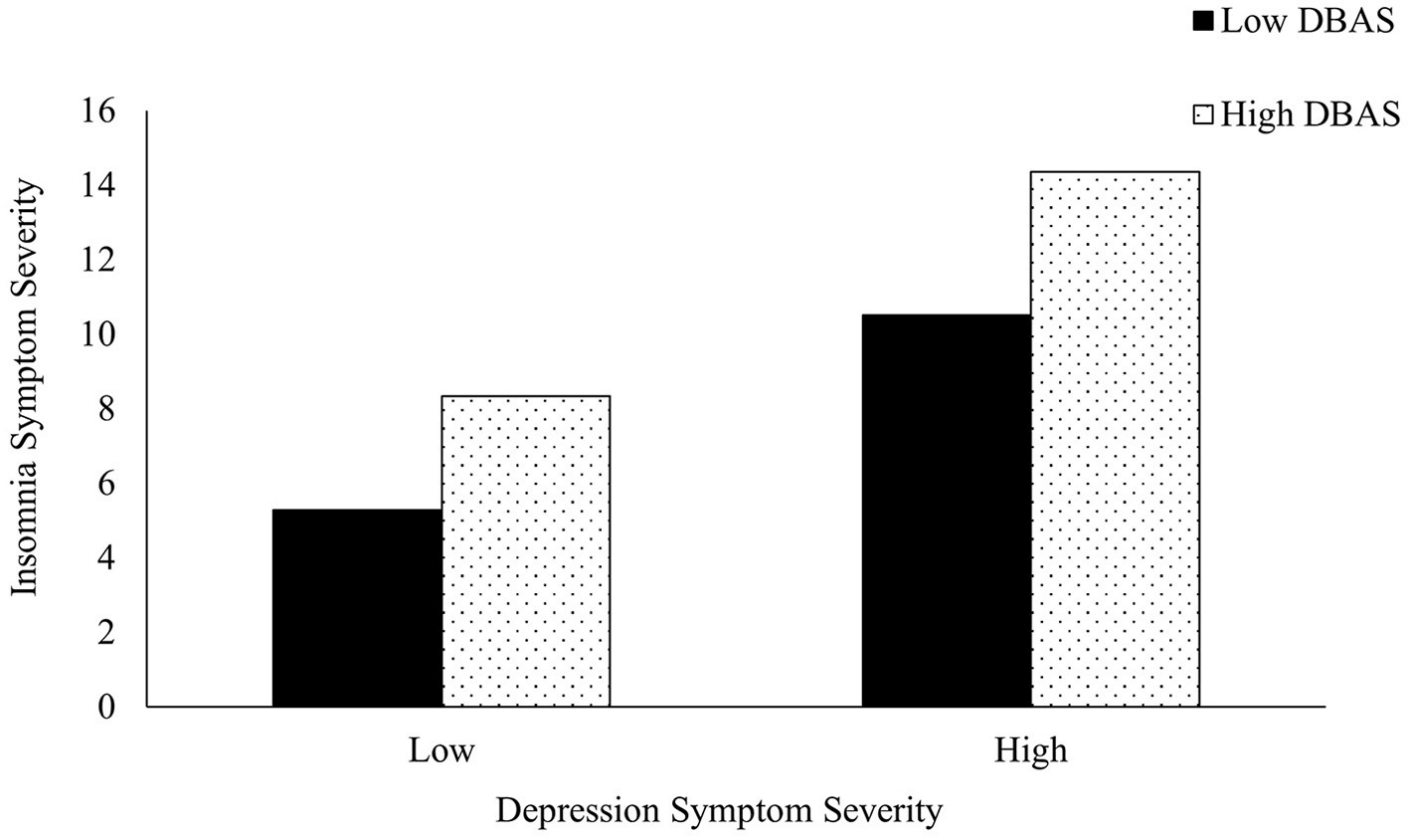
The moderating effect of dysfunctional beliefs and attitudes about sleep (DBAS) on the association between depression and insomnia symptom severity. Low symptom severity represents “none/minimal” depressive symptoms, while high symptom severity represents “moderately severe” depressive symptoms according to the PHQ.

**Figure 2:**
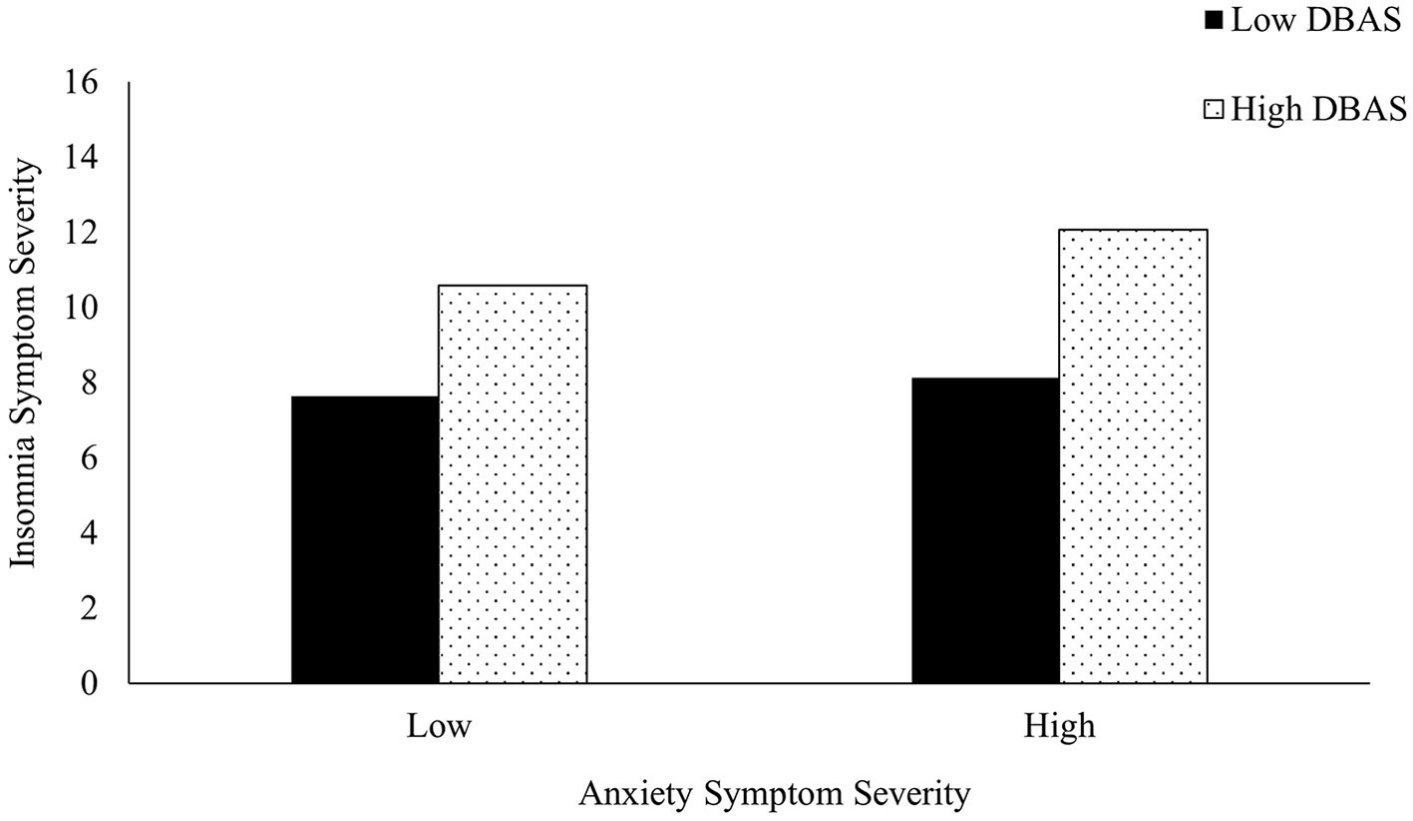
The moderating effect of dysfunctional beliefs and attitudes about sleep (DBAS) on the association between anxiety and insomnia symptom severity. Low symptom severity represents “minimal” anxiety symptoms, while high symptom severity represents “moderately severe” anxiety symptoms according to the GAD-7.

Dysfunctional sleep beliefs also significantly predicted poor sleep outcomes [*R*^2^ = 0.315, *F*_(5,1,508)_ = 17.990, *b* = 1.963, *p* < 0.001] and moderated the association between depressive symptoms (*p* =0.035), anxiety symptoms (*p* =0.007), and sleep (Table 4). High dysfunctional sleep beliefs exacerbated the impact of poor mental health on sleep and were associated with more severe insomnia symptoms compared to low dysfunctional sleep beliefs (Figures 1, 2). This was especially true for those with low levels of depression and anxiety. Specifically, low depressive or anxiety symptoms and low DBAS were not associated with clinical insomnia symptoms; however, low depressive or anxiety symptoms and high DBAS were associated with subthreshold insomnia, as characterized by the ISI. Regardless of the level of depressive and anxiety symptoms, higher dysfunctional sleep beliefs were associated with more severe insomnia symptoms compared to those with lower dysfunctional sleep beliefs. Moderation models did not differ between students identifying as white vs. POC for depression and perceived stress, however; dysfunctional sleep beliefs significantly moderated the relation between anxiety and insomnia severity in POC (*b* = 0.031, 95% CI = 0.004 to 0.058, *p* = 0.026) but not white students (*b* = 0.028, 95% CI = −0.007 to 0.063, *p* = 0.115).

**Table 4:** Regression coefficients of the moderating effect of dysfunctional sleep beliefs (DBAS) on insomnia severity and mental health outcomes.

**DV**		***R^2^***	***b***	***SE b***	***95% CIs***	***p***
ISI	Depression	0.566	0.347	0.0542	(0.240, 0.453)	< 0.001
	DBAS		0.945	0.122	(0.706, 1.184)	< 0.001
	Interaction		0.020	0.010	(0.001, 0.039)	0.035
	Anxiety	0.567	−0.053	0.060	(−0.171, 0.066)	0.383
	DBAS		0.867	0.127	(0.618, 1.116)	< 0.001
	Interaction		0.029	0.011	(0.008, 0.051)	0.007
	Perceived stress	0.566	−0.057	0.048	(−0.152, 0.038)	0.239
	DBAS		0.801	0.213	(0.384, 1.218)	< 0.001
	Interaction		0.017	0.009	(−0.002, 0.035)	0.079

Sex, income, psychiatric diagnosis, race/ethnicity, and unexamined mental health outcomes were used as covariates.

ISI, insomnia severity index; DBAS, dysfunctional beliefs and attitudes about sleep.

## 4 Discussion

Over half of the 1,562 students surveyed in this study reported moderate-to-severe stress levels and insomnia symptoms. The findings from the current study are worrisome given 42% of students met the cut-off for severe symptoms of depression or anxiety. Higher dysfunctional sleep beliefs were independently associated with poorer mental health and sleep outcomes, which replicates previous findings (Peng et al., 2023). Overall, our results reaffirm prior claims that poor mental health can lead to maladaptive sleeping behaviors (Nutt et al., 2008; Ramsawh et al., 2009; Hamilton et al., 2021), and reveal a novel and important contribution of dysfunctional sleep beliefs as a factor that exacerbates this association. To our knowledge, this is the first study to suggest the moderating effect of dysfunctional beliefs on sleep through mental health in university students.

In this study, reporting higher dysfunctional sleep beliefs was related to having worse depression, anxiety, perceived stress, and insomnia symptoms. A novel finding is that higher dysfunctional sleep beliefs potentiated the association between poor mental health and sleep in university students. This is important for several reasons. First, young adulthood is a developmental period marred by novel stressors associated with school and life (Shaw et al., 2017; Reddy et al., 2018), and some young adults may lack the coping skills needed to deal with such stressors (Morin et al., 2002). Second, mental illness tends to emerge during this developmental period (Kessler et al., 2007; Jones, 2013) and the preliminary results here suggest that dysfunctional sleep beliefs may strengthen the association between poor mental health and poor sleep, though our results need to be followed with longitudinal research. Third, these findings are especially important since dysfunctional sleep beliefs can manifest similarly to depression and anxiety and students may be unaware that dysfunctional sleep beliefs are impacting their mental health and sleep simultaneously. Finally, POC made up over half of the respondents surveyed and they reported having higher dysfunctional sleep beliefs and insomnia severity than white students. Although we did not find race/ethnicity to significantly predict worse mental health when other factors were accounted for, we did find that dysfunctional sleep beliefs moderated the relationship between anxiety and insomnia for POC students but not for white students. Specifically, POC with higher anxiety and higher dysfunctional sleep beliefs were associated with worse symptoms of insomnia. Anxiety can disrupt sleep (Manzar et al., 2021), and when it co-occurs with minority stressors as well as cultural differences in perceptions and attitudes toward inadequate sleep (Shangani et al., 2020; Cheung et al., 2021; Jeon et al., 2021), it may be more likely to cause disordered sleep (Bermudez et al., 2022). Educating students about sleep beliefs and sleep hygiene may be critical for improving sleep habits and mental health outcomes. Kloss et al. (2016) demonstrated that an educational sleep program, “Sleep 101,” improved maladaptive sleep beliefs among post-secondary students and, in turn, improved sleep quantity. Future studies should examine bridging the gap in sleep knowledge among students, particularly the presentation and impact of dysfunctional sleep beliefs.

Since dysfunctional sleep beliefs may exacerbate mental health issues and poor sleep, it is important to understand how these beliefs arise so that steps can be taken to combat them. A study on recent internet searches suggests that many people have limited knowledge about sleep behaviors, which contributes to distorted sleep beliefs and can influence their sleeping patterns (Robbins et al., 2019). For example, participants believed the myth that “many adults need only 5 h or less of sleep for general health,” which is substantially lower than the recommended guideline of seven to nine hours per night (Hirshkowitz et al., 2015). Another source of dysfunctional sleep beliefs may be the ubiquitous health monitoring applications or smartwatches that track sleep and provide feedback as an overall “sleep score.” Approximately 30% of Canadians use wearable devices to track sleep patterns (Paré et al., 2018). Unfortunately, one's sleep score may not be a true representation of actual sleep, as these devices have tremendous variability associated with the estimates used to calculate the sleep score (Stone et al., 2020). Nonetheless, individuals who analyze their wearable sleep data tend to get overly concerned with improving or perfecting their sleep due to sleep myths such as, “One night of sleep deprivation will have lasting negative health consequences” (Baron et al., 2017; Robbins et al., 2019). Individuals may also misinterpret the metrics wearables report, such as “deep sleep” or sleep duration. For example, individuals may believe they are achieving sufficient sleep when sleeping for the recommended seven to nine hours, but may still feel unrested if they experience frequent awakenings at night. The misalignment between actual versus estimated sleep scores may fuel maladaptive cognitions and poor sleep beliefs. Previous research has demonstrated that students can be susceptible to placebo/nocebo sleep effects (Draganich and Erdal, 2014). That is, participants who slept well but received deceptive feedback indicating poor sleep performed worse on subsequent cognitive tasks of attention and processing speed. Importantly, accessible education about sleep beliefs and behaviors can help students reframe their cognitions about sleep to minimize these discrepancies and improve their mental health and sleeping patterns.

Although dysfunctional sleep beliefs moderated the relationship between depression and anxiety with insomnia, dysfunctional sleep beliefs did not moderate the relationship between perceived stress and insomnia. Given that insomnia symptoms can arise from a multitude of stressors including physiological, psychological, and environmental issues and their compounding effects (Levenson et al., 2015), it may be that dysfunctional sleep beliefs are not a moderator but a mediator or mechanism through which perceived stress causes insomnia (Brand et al., 2010). However, because this was a cross-sectional study, we did not explore mediating mechanisms but would encourage future interventions to do so.

### 4.1 Strengths, limitations, and future directions

Our sample represents the Canadian university population by average age (M = 21.8, SD = 4.8) and race/ethnicity. Statistics Canada (2021) reported that ~60% of students enrolled in post-secondary education were visible minorities. Our study reflects this diversity, with 58% of participants identifying as POC across the two universities. However, our study had a greater proportion of females compared to the broader Canadian post-secondary population (Statistics Canada, 2021). This likely reflects the observation that females are more likely to participate in survey-based studies (Nuzzo, 2021).

Although the current study had a large sample size, the cross-sectional nature only provides a snapshot of self-reported conditions and cannot provide evidence for causality in the associations between dysfunctional sleep beliefs, mental health, and sleep. As such, future longitudinal research is needed, followed by interventions and randomized control trials to test whether changes in dysfunctional sleep beliefs and mental health cause changes in sleep patterns. Further, our sample may not be representative of all students as recruitment only occurred at two Ontario universities. The current study was advertised as a sleep study, which may have unintentionally introduced a sampling bias. Individuals who are interested in sleep or have poorer sleep may have been more inclined to participate in the current study. This potential self-selection bias could distort the findings by overrepresenting individuals with sleep-related concerns or issues.

Additionally, since this was a survey-based study, there were no objective sleep measures and it is possible that mental health states could have impacted self-perceptions of sleep and stress. As such, individuals may have had objectively better sleep or stress levels but perceived it as worse due to poor mental health (Stremler et al., 2020). It is also important to note that this data took a combined racial and ethnic lens and used a binary categorization of white and POC. Future research is encouraged to investigate both constructs with greater variance to see how differences in race (e.g., physical characteristics) and ethnicity (e.g., religion, culture, and geographical region) independently and collaboratively relate to both mental health and sleep outcomes and the underlying mechanisms driving these relationships. Learning more about differences in sleep, mental health, and stress outcomes across different identities (e.g., gender, sexuality, disability, etc.) and their intersections is an important step for future research. This can be done by intentionally sampling from more diverse groups and building relationships with targeted offices and student groups on university campuses.

## 5 Conclusion

To our knowledge, this is the first study to examine the interplay between dysfunctional sleep beliefs and mental health on sleep in university students. While it has been previously shown that dysfunctional sleep beliefs negatively impact mental health and sleep independently (Jansson-Fröjmark and Linton, 2008; Eidelman et al., 2016; Chang et al., 2020), the current study provides an explanatory model that points to dysfunctional sleep beliefs as a potential target for interventions. Critically, the results suggest that dysfunctional sleep beliefs may have serious health-compromising effects, elevating symptoms of poor mental health to exacerbate poor sleep outcomes. More accessible supports to shift dysfunctional sleep beliefs among students to help mitigate poor sleep and promote mental health are needed.

## Data availability statement

The raw data supporting the conclusions of this article will be made available by the authors, without undue reservation.

## Ethics statement

The studies involving humans were approved by McMaster Research Ethics Board and Office of Research Ethics—University of Waterloo. The studies were conducted in accordance with the local legislation and institutional requirements. The participants provided their written informed consent to participate in this study.

## Author contributions

SK: Conceptualization, Data curation, Formal analysis, Project administration, Writing – original draft, Writing – review & editing. TK: Conceptualization, Data curation, Methodology, Project administration, Validation, Writing – review & editing. MO: Writing – review & editing. LM: Conceptualization, Methodology, Supervision, Validation, Writing – review & editing. JH: Conceptualization, Formal analysis, Methodology, Supervision, Validation, Writing – review & editing.
